# Investigation of the Etiology of Molar Incisor Hypomineralization in Children Residing in Konya Province and Surrounding Areas, Türkiye

**DOI:** 10.3390/children11111399

**Published:** 2024-11-19

**Authors:** Aslı Seloğlu, Firdevs Kahvecioğlu

**Affiliations:** 1 Beyhekim Oral and Dental Health Center, 42040 Konya, Türkiye; asliseloglu@gmail.com; 2 Department of Pedodontics, Faculty of Dentistry, Selçuk University, 42250 Konya, Türkiye

**Keywords:** molar incisor hypomineralization, etiology, questionnaire

## Abstract

Background: According to the literature, hypomineralization of molars and incisors is a multifactorial condition that depends on both genetic and environmental factors. This study aims to diagnose Molar Incisor Hypomineralization (MIH) cases with a new index that better defines the defect and to contribute to the literature by identifying possible etiological factors. Methods: This research was conducted with children aged 8–11 years old and their parents from Konya province and surrounding provinces. While children who were diagnosed with MIH as a result of the examination constituted the study group, children with no findings of MIH during intraoral examination were included in the control group. Between February and October 2020, 104 patients for the study group and 104 patients for the control group were reached, and a survey was administered to a total of 208 parents. Results: Asthma, pneumonia, lower respiratory tract infections, diarrhea, a fever, and febrile convulsions between the ages of 0–4 have been found to be related to MIH (*p* < 0.05). Conclusions: The association between medical diseases in children and enamel defect formation draws attention to the importance of pediatricians in the early diagnosis of MIH cases. Pediatricians can be very helpful in informing parents of children with health problems about possible dental defects and referring them to a pediatric dentist.

## 1. Introduction

Tooth enamel, unlike other hard tissues in the human body, does not have the ability to remodel. For this reason, physical changes that occur during enamel formation occur permanently on the tooth surface [1]. Because ameloblasts are sensitive to environmental changes, they are affected by even minor physiological changes during enamel formation. While minor changes cause damage that can only be observed at the histological level, more severe damage produces results that can be observed clinically [2]. In addition, the severity of the agent causing the enamel defect, its duration, and the activity level of the ameloblasts at the time of exposure to the agent also affect the degree of the defect [3]. While disturbances in the secretion phase reduce the amount of enamel and cause enamel hypoplasia, disturbances in the maturation phase affect the enamel quality and cause the formation of hypomineralized enamel, which has an opaque appearance [1,4].

Developmental enamel defects can have many different clinical characteristics, ranging from a complete absence of tooth enamel to widespread enamel opacities. When developmental enamel defects are classified, regardless of their etiology, they are observed as defects, including limited opacities, widespread opacities, enamel hypoplasia, and a combination of these. There are three conditions generally characterized by developmental enamel defects. These are amelogenesis imperfecta, which is a condition in which the proteins involved in enamel production are affected by genetic variations; fluorosis, which occurs due to excessive fluoride intake during enamel formation; and a condition called molar incisor hypomineralization (MIH), which is thought to be caused by a systemic imbalance, but the exact cause has not yet been determined [4].

The term MIH refers to a condition whose etiology is still unknown but is thought to be of systemic origin, affecting one or more permanent first molars and maybe also affecting permanent incisors [5].

According to the current literature, MIH is a qualitative defect resulting from a dysfunction in ameloblasts in the late stages of the mineralization phase of enamel formation [4]. Rapidly developing caries, atypical restorations, and tooth extraction due to MIH are common in teeth affected by MIH [6]. Additionally, de Aguiar Grossi et al. [7] reported that MIH was a risk factor for the development of caries in a systematic review. Due to all these features, MIH is distinguished from amelogenesis imperfecta, a genetic disease with generalized enamel defects, fluorosis, a generalized enamel defect, and localized enamel defects caused by primary tooth infection or trauma [5].

According to many studies conducted on the prevalence of MIH in different parts of the world and the newly published systematic review, the lowest prevalence of MIH was found in Chinese children at 2.8%, the highest prevalence of MIH was found in Brazilian children at 40.2%, and the prevalence of MIH worldwide was found to be 14.2%; South America (18.0%) and Spain (21.1%) had the highest prevalence values [4,8,9,10].

When the current literature on the etiology of MIH is examined, we can examine the predisposing factors that play a role in the development of MIH under three main headings; prenatal, perinatal, and postnatal factors. Prenatal factors include maternal fever attacks, viral infections in the last month of pregnancy, and long-term drug therapy [11,12]. Perinatal factors are birth complications, premature birth, and low birth weight [12,13]. As postnatal factors of MIH etiology in the literature, malnutrition, gastroenteritis, respiratory tract infections, pneumonia, asthma, high fever, febrile convulsion, childhood diseases, urinary tract infections, middle ear infections, and antibiotic use are shown [13,14,15,16,17,18].

According to the literature, MIH is a multifactorial clinical condition that depends on both genetic and environmental factors [13]. It is known that enamel production is controlled by various genes. Although it is thought that a genetic variation in these genes may cause MIH, more studies are needed on this subject [19]. In this context, this study aims to diagnose MIH cases with a new index that better defines the defect and contributes to the literature by determining possible etiological factors. In this thesis, a study was carried out for this purpose; patients living in and around Konya province (a region that has not been examined before in terms of the etiology of MIH) who applied to the Department of Pedodontics of Selçuk University Faculty of Dentistry for examination were reviewed with an updated index for the diagnosis of MIH, and we tried to determine possible etiological factors.

## 2. Materials and Methods

### 2.1. Population and Sample

This study was designed as an explanatory case-control study. The study was conducted with children aged 8–11 years old and their parents from Konya province and surrounding provinces who applied to the Department of Pedodontics of Selçuk University Faculty of Dentistry for examination in 2020. While children diagnosed with MIH as a result of the examination constituted the study group, children with no findings of MIH during intraoral examination were included in the control group. The study examined data from the surveys administered to the parents of children in both groups.

Power analysis was performed to determine the sample size of the study and control groups. A comprehensive literature review was conducted to determine the effect size, and (f) was determined to be 0.50. In order to avoid type 2 errors, the power value was taken as 0.95. Under these conditions, the number of patients determined using the GPower 3.1.9.2 program was 104 for each group. Between February 2020 and October 2020, 104 patients from the study group and 104 patients from the control group were contacted, and a survey was administered to a total of 208 parents.

The clinical examination was performed by two permanent observers. Intra-examiner calibration was checked by repeating examinations of 20 children after an interval of 5 days. The inter-examiner reliability (Cohen’s kappa) statistics of the two examiners was 0.93, and the examiner consistency (intra-examiner) was 0.95.

### 2.2. Ethical Consideration

Ethics committee approval numbered 2020/05 was received for the research from Selçuk University Faculty of Dentistry Non-Interventional Scientific Research Evaluation Commission on 13 February 2020.

### 2.3. Intraoral Examination

Intraoral examination was performed while the participants’ teeth were moist after oral hygiene was achieved. All surfaces of the teeth were examined under adequate illumination with the help of a mirror and a probe.

The teeth examined in the mouth were permanent incisors and permanent first molars. All surfaces of the examined teeth were examined, and if there was more than one defect on different surfaces of the same tooth, the most severe one was taken into account. In the children in the study and control groups, at least half of the permanent first molars were required to have erupted in the mouth. In order to be diagnosed with MIH, it was taken into account that at least one of the permanent first molars must be affected. The examination of the children evaluated lasted approximately 3 min per child. An index record containing Ghanim et al.’s diagnostic criteria for MIH was used (Table 1) [20]. According to this index, each tooth evaluated for the diagnosis of MIH was given a value between 0 and 10.

**Figure 1:**
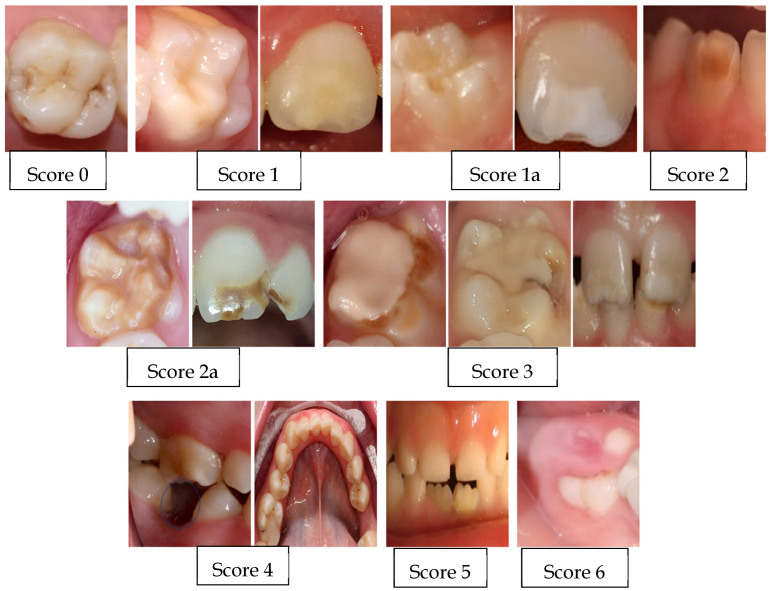
Examination images and the number of scores.

If there were no defects in the enamel of the evaluated first molars and incisors that met the MIH criteria, a value of ‘0’ was given. If the presence of caries and restoration in these teeth was not related to MIH, the defect was considered to be absent. Children between the ages of 8–11 with teeth given the value ‘0’ were included in the control group. White/cream-colored opacities with well-defined borders, without loss of substance after application, were given the value ‘1’ in the index and were included in the study group. White/cream-colored opacities with well-defined borders with loss of substance after application were given the value ‘1a’ in the index and were included in the study group. Yellow/brown opacities with well-defined borders, without loss of substance after application, were given the value ‘2’ in the index and were included in the study group. Yellow/brown opacities with well-defined borders with loss of substance after application were given the value ‘2a’ in the index and were included in the study group. In first molars, restorations that did not follow the boundaries of the caries cavity and generally extended to buccal or lingual/palatinal flat surfaces and atypical buccal restorations in incisors without a history of dental trauma were considered to be associated with MIH and were included in the study group by receiving the value ‘3’ in the index. Tooth loss thought to have occurred due to MIH was also included in the evaluation. It has been stated that in a case where the first molar is thought to be extracted due to MIH (Molar Incisor Hypomineralization), other permanent first molars in the mouth may also show signs of MIH. These teeth were included in the study group by receiving the value ‘4’ in the index. Partially erupted teeth with the appearance of MIH were considered suspicious for the diagnosis of MIH and, therefore, were excluded from evaluation and were not included in the study or control group. These teeth were given the value ‘5’ in the index. Partially erupted or impacted teeth with no evidence of MIH were also excluded from evaluation by not being included in the study or control groups. These teeth were given a score of ‘6’ in the index. Cases with diffuse opacities in all teeth, such as fluorosis or amelogenesis imperfecta, were not included in the study group because they were not suitable for MIH. Since it is thought that many genetic, environmental, and systemic factors may cause such a situation, these teeth were excluded from the study by not being included in the control group to make the study data transparent. These teeth were given a score of ‘7’ in the index. Enamel hypoplasia is a condition in which any local, systemic, or genetic malfunction in the secretory phase of enamel formation results in a decrease in enamel thickness by affecting the enamel organic matrix content and normal ameloblast morphology and physiology. Hypoplasia cases, which have a different clinical picture with MIH, were excluded from the evaluation by not being included in the study and control groups. These teeth were given the value ‘8’ in the index. Since this study is only about the etiology of the clinical condition MIH, combined cases in which MIH is accompanied by hypoplasia and diffuse opacities were not included in the study, nor in the control group. These teeth were given the value ‘9’ in the index. These cases were not included in the MIH group due to the possibility that only the opacities with well-defined borders in the incisors, detected in the intraoral examination, occurred as a result of local factors. These teeth were given the value ‘10’ in the index. The examination images and number of scores are given in Figure 1.

### 2.4. Etiological Evaluation

A comprehensive questionnaire was administered to the parents of all children in the study, including all etiological factors suggested to cause MIH in the literature. To ensure the reliability of the survey questions, a pilot study with n = 30 samples was conducted. An acceptable reliability coefficient was reached (item extracted Cronbach alpha = 0.608). The questions in the survey form are listed in Table 2 with their main headings.

## 3. Statistical Analysis

Data compiled through face-to-face interviews were digitized. IBM SPSS “Statistical Package for the Social Sciences” 26.0 was used for statistical analysis in the study. Firstly, Binary Logistic Regression analysis was applied to examine the influence between independent variables and MIH. Logistic Regression assumptions were met (max_VIF_ = 1.191 and max_TOLERANCE_ = 0.962); however, the model was not found to be significant (*p* = 0.333). Thereupon, the chi-square tests were applied to examine the changes in independent variables based on the study and control groups. Frequencies, percentages, and chi-square significance are given based on groups. Odds ratios were also given, along with their significance. A level of 0.05 was accepted as significant in all statistical tests within the study.

## 4. Results

Participants were between 8 and 11 years old. The mean age was 9.5 years, and the standard deviation was 1.12. A total of 51.9% of the study group was female, and 54.8% of the control group was male. Chi-square analyses showing the association between various variables and MIH are demonstrated in Table 3.

According to the chi-square and odds ratio analysis results, although the rate of those who had a disease or birth complication in the last 3 months of pregnancy was higher in the study group than in the control group, no statistically significant difference was detected (*p* > 0.05). When the groups were compared in terms of preterm birth, it was seen that there was no significant difference between them (*p* > 0.05). When compared in terms of birth weights, no significant difference was found between groups (*p* > 0.05). When the children were compared in terms of breastfeeding duration, there was no statistically significant difference between groups (*p* > 0.05). No statistically significant difference existed between groups in terms of using fluoride, calcium, and multivitamin tablets (*p* > 0.05). There was a statistically significant difference between groups in terms of having diarrhea (*p* < 0.05). This is also supported by a significant odds ratio of 3.08. The incidence of severe diarrhea was 13.5 percent for the study group and 4.8 percent for the control group. There was no statistically significant difference between groups (*p* > 0.05). A statistically significant difference existed between groups in terms of having asthma (*p* < 0.05). The incidence of asthma was 12.5 percent for the study group and 2.9 percent for the control group, and the odds ratio was 4.81. There was a statistically significant difference between groups in terms of having pneumonia and lower respiratory tract infections (*p* < 0.05). The incidence of lower respiratory tract infections was 30.8 percent for the study group and 18.3 percent for the control group. Odds ratios were 2.75 for pneumonia and 1.99 for lower respiratory tract infections, respectively. There was a statistically significant difference between groups in terms of having seizures (*p* < 0.05). The incidence of seizures was 12.5 percent for the study group and 4.8 percent for the control group, and the odds ratio was 2.83. A statistically significant difference existed between groups in terms of having frequent fever (*p* < 0.05). The incidence of frequent fevers was 61.5 percent for the study group and 47.1 percent for the control group, and the odds ratio was 1.80. No statistically significant difference existed between groups in terms of having a middle-ear infection (*p* > 0.05). It was found that there was no statistically significant difference between groups in terms of having kidney and urinary tract disorders (*p* > 0.05). When the children in the study and control groups were compared in terms of childhood illnesses (measles, rubella, scarlet fever, chickenpox, mumps), no statistically significant difference was found between groups (*p* > 0.05).

## 5. Discussion

Research on the etiology of MIH takes into account the onset and completion times of mineralization of permanent first molars [21]. Crown calcifications of permanent first molars begin at birth or just before birth and are completed at the age of 4–5 [22,23]. For this reason, questions regarding the anamnesis of the children included in our study cover the prenatal period, the moment of birth, and the first 4 years of life.

Birth complications such as cesarean section may pose a risk for MIH as they may cause hypoxia [24]. Silva et al. [13] emphasized that maternal disease and birth complications may be associated with MIH. On the other hand, there are also studies that do not find prenatal factors and birth complications to be related to MIH [15,25,26,27,28,29]. In this study, no difference was reported between the control and study groups regarding prenatal factors and birth complications (*p* = 0.0775).

There are studies showing the association between premature birth and low birth weight and MIH [27,30,31]. However, there are also studies that do not indicate association [28,32]. In their meta-analysis, Fatturi et al. [33] stated that studies finding an association between premature birth, duration of breastfeeding, low birth weight, and MIH are in the minority. Consistent with these data, it was also observed in our study that there was no difference between the study and control groups in terms of premature birth, duration of breastfeeding, and birth weight (*p* = 0.206) (*p* = 0.593) (*p* = 0.818).

In our study, no difference was found between fluoride intake and MIH formation. These data are consistent with studies stating that fluoride is not associated with the etiology of MIH [8]. Fluoride is responsible for diffuse linear opacities in homologous teeth, which is inconsistent with MIH [4].

Although no difference was found between the intake of calcium supplements and the etiology of MIH in our study, the percentage of MIH in those with frequent diarrhea was found to be statistically significant. This finding aligns with the data reported by Sönmez et al. [15], who noted that frequent diarrhea during the first 4 years of life can lead to deficiencies in phosphorus and vitamins A, C, and D—nutrients crucial for tooth development—as well as calcium. This may help explain the MIH situation that occurred in the children in our study who had frequent diarrhea despite taking multivitamin supplements. However, more research is needed to reveal any direct association.

Among the postnatal etiological factors, respiratory diseases have been associated with MIH [29,33,34,35]. Hypoventilation, seen in upper respiratory tract diseases such as asthma and adenoid infections and lower respiratory tract diseases such as pneumonia and bronchitis, can affect the enamel matrix pH by causing respiratory acidosis with abnormal oxygen levels. Increased acidity in the enamel matrix leads to enamel hypomineralization by inhibiting proteolytic enzyme activity [28].

Asthma is one of the diseases frequently seen in children diagnosed with MIH [24,28,36]. It is thought that the impaired protein matrix structure, which occurs due to lack of oxygen in patients with asthma problems, causes hypomineralization [28,32]. In addition, cortico-steroid drugs used in the treatment of asthma negatively affect bone growth by affecting osteoblasts [37]. These drugs may also have the same effect on ameloblasts, causing enamel hypoplasia and hypomineralization [38]. In contrast to these data supporting the association between asthma and MIH, Hoffmann et al. [39] did not find an association between asthma and MIH. The possible reason for this may be that the sample size evaluated was smaller compared to other studies. In our study, asthma experienced in the first 4 years of life was found to be associated with MIH.

In this study, it was observed that the number of patients with febrile convulsions was statistically significantly higher in the study group than in the control group (*p* = 0.049). This result is consistent with studies showing that febrile convulsions and recurrent high fever are associated with MIH [25,29,35,36]. Frequent fever attacks can lead to the formation of MIH by affecting the ameloblastic activity and causing degeneration in enamel prisms [15,30,31]. These data explain the significantly higher number of people with frequent fevers in the 0–4 age range in our study group (*p* = 0.037).

There are a limited number of studies finding associations between otitis media and MIH [25,40]. In this study, no significant difference was found between children who had otitis media in the first 4 years of life and the occurrence of MIH (*p* = 0.664).

Kuscu et al. [41] found a significant association between kidney diseases and MIH. There are a few other studies that demonstrated the same association [26], but it was stated in meta-analysis studies that there were not enough studies to support this [13]. While Jalevik et al. [25] found a significant association between urinary tract infections and MIH in their study, Allazzam et al. [28] found no association. According to the data obtained from our study, no significant difference was detected between both kidney diseases and urinary tract infections and MIH (*p* = 0.999).

Although older studies in the literature found an association between childhood rash diseases and MIH, a meta-analysis study by Fatturi et al. [33] found no association between childhood diseases and MIH. The present study found no statistically significant association between measles, rubella, scarlet fever, mumps, and chickenpox and the incidence of MIH. It is known that the incidence of these childhood diseases in today’s population has decreased thanks to vaccination. Despite the decrease in disease incidence, the increase in the prevalence of MIH [10] explains the lack of a significant association between childhood diseases and MIH.

Our study, in line with the findings of previous similar studies, showed that different environmental and medical factors may be related to MIH. This result seems logical because enamel formation is a long and sensitive period and can be affected by many environmental stimuli. In addition, many medical conditions are likely to occur during childhood, so it is unlikely that a single factor is responsible for the etiology of MIH. The association between medical diseases in children and defective enamel formation draws attention to the importance of pediatricians in the early diagnosis of MIH cases. Because families of children with health problems prioritize medical treatment over their dental health, pediatricians can be very helpful in informing parents about possible dental defects and referring them to a pediatric dentist.

There are many studies on the etiology of MIH, but since these studies are generally based on parents’ recollections of a situation years ago, they have some limitations, and parents may give biased answers to the questions. In order to minimize biased answers, by conducting comprehensive questioning, parents were not given any information about the effect of the investigated disease on the formation of MIH. Although our method reduces biased answers, it is impossible to fully learn the child’s health status in the first 4 years of life. To reach more accurate and precise results, benefitting from the data recorded by health centers may be preferable. Since it is impossible to reach these data through retrospective studies for every patient, prospective planning of studies on the etiology of MIH would be a solution.

This study was conducted on a group of 208 children. A significant association was indicated between some of the possible etiological factors and MIH. We think that our study should be supported by prospective observational studies involving larger populations to fully explain the factors responsible for the etiology of MIH.

## 6. Conclusions

- It has been observed that prenatal factors such as maternal diseases experienced in the last 3 months of pregnancy and perinatal factors such as premature birth and birth complications are not associated with MIH.
- The duration of breastfeeding and fluoride, calcium, and vitamin supplements taken in the first 4 years of life have no association with MIH.
- It has been determined that frequent diarrhea and fever attacks in the first 4 years of life, febrile convulsions, asthma, pneumonia, and lower respiratory tract diseases have been observed to be associated with MIH.
- It has been observed that childhood diseases with rashes, kidney diseases, and urinary tract infections are not associated with MIH.

## Figures and Tables

**Table 1:** Criteria for scoring molar incisor hypomineralization according to European Academy of Paediatric Dentistry recommendations [20], copyright permission is taken from Wiley Online Library.

0	Enamel defect free
1	White/creamy demarcated opacities, no PEB
1a	White/creamy demarcated opacities, with PEB
2	Yellow/brown demarcated opacities, no PEB
2a	Yellow/brown demarcated opacities, with PEB
3	Atypical restoration
4	Missing because of MIH
5	Partially erupted (i.e., less than one-third of the crown high) with evidence of MIH
6	Unerupted/partially erupted with no evidence of MIH
7	Diffuse opacities (not MIH)
8	Hypoplasia (not MIH)
9	Combined lesion (diffuse opacities/hypoplasia with MIH)
10	Demarcated opacities in incisors only

MIH, molar incisor hypomineralization; PEB, post-eruptive enamel breakdown.

**Table 2:** Survey form questions with main headings.

Prenatal Factors
Whether the mother had any disease in the last 3 months of her pregnancy; if so, the type of disease
Perinatal Factors
Whether complications occurred at the moment of birth; if so, the type of complication Whether the child was born prematurely; if so, the week in which the baby was delivered Birth weight of the child (less than 1.5 kg/between 1.5–2.5 kg/more than 2.5 kg)
Postnatal Factors
Duration of breastfeeding the child (never/less than 8 months/between 8–12 months/more than 12 months) Whether the child was given fluoride, calcium, or vitamin supplements before the age of 4 Whether the child had a digestive system disease until the age of 4 Whether the child had a respiratory system disease until the age of 4 (such as asthma, pneumonia, bronchitis, bronchiolitis, laryngitis, tonsillitis, pharyngitis, angina) Whether the child had a fever and febrile convulsions (seizures) until the age of 4 Whether the child had otitis media (middle-ear infection) until the age of 4 Whether the child had a urinary tract infection and nephropathy until the age of 4 What childhood rash diseases the child had until the age of 4

**Table 3:** Association between various variables and MIH.

		Group n (%)		X^2^	*p*	Odds Ratio (%95 L-U)
		Control	Study			
Did you have any illnesses or birth complications in the last 3 months of your pregnancy?	Yes	6 (5.8)	7 (6.7)	0.082	0.775	1.179 (0.382–3.634)
	No	98 (94.2)	97 (93.3)			
Was your child born prematurely?	Yes	6 (5.8)	11 (10.6)	1.601	0.206	1.932 (0.687–5.435)
	No	98 (94.2)	93 (89.4)			
What was your child’s birth weight?	1.5–2.5 kg	10 (9.6)	11 (10.6)	0.053	0.818	1.112 (0.451–2.743)
	>2.5 kg	94 (90.4)	93 (89.4)			
How long did you breastfeed your child?	>12 months	20 (19.2)	17 (16.3)	1.992	0.574	-
	8–12 months	40 (38.5)	50 (48.1)			
	<8 months	44 (42.3)	37 (35.6)			
Did you give your child fluoride tablets, calcium tablets, or vitamin tablets before the age of 4?	Yes	34 (32.7)	30 (28.8)	0.361	0.548	0.835 (0.463–1.505)
	No	70 (67.3)	74 (71.2)			
Did your child have severe diarrhea by the age of 4?	Yes	5 (4.8)	14 (13.5)	4.692	*0.030* *	*3.080* * (1.067–8.892)
	No	99 (95.2)	90 (86.5)			
Did your child have any digestive problems until the age of 4?	Yes	3 (2.9)	3 (2.9)	0.000	0.999	1.000 (0.197–5.073)
	No	101(97.1)	101 (97.1)			
Did your child have asthma since birth until the age of 4?	Yes	3 (2.9)	13 (12.5)	6.771	*0.009* *	*4.810* * (1.328–17.419)
	No	101 (97.1)	91 (87.5)			
Did your child have pneumonia by the age of 4?	Yes	6 (5.8)	15 (14.4)	4.290	*0.038* *	*2.753* * (1.024–7.403)
	No	98 (94.2)	89 (85.6)			
Did your child have lower respiratory tract infections such as bronchitis, bronchiolitis, and laryngitis until the age of 4?	Yes	19 (18.3)	32 (30.8)	4.390	*0.036* *	*1.988* * (1.039–3.804)
	No	85 (81.7)	72 (69.2)			
Did your child have seizures by the age of 4?	Yes	5 (4.8)	13 (12.5)	3.892	*0.049* *	*2.829* * (1.007–8.246)
	No	99 (95.2)	91 (87.5)			
Did your child have frequent fevers until the age of 4?	Yes	49 (47.1)	64 (61.5)	4.360	*0.037* *	*1.796* * (1.034–3.118)
	No	55 (52.9)	40 (38.5)			
Did your child have a middle-ear infection by the age of 4?	Yes	13 (12.5)	11 (10.6)	0.188	0.664	0.828 (0.353–1.944)
	No	91 (87.5)	93 (89.4)			
Did your child have chronic kidney failure or another kidney disease until the age of 4?	Yes	4 (3.8)	4 (3.8)	0.000	0.999	1.000 (0.243–4.110)
	No	100 (96.2)	100 (96.2)			
Did your child have a urinary tract infection by the age of 4?	Yes	18 (17.3)	18 (17.3)	0.000	0.999	1.000 (0.488–2.051)
	No	86 (82.7)	86 (82.7)			
What childhood illnesses did your child have until the age of 4?	None	76 (73.1)	73 (70.2)	0.307	0.858	-
	Chick pox	20 (19.2)	21 (20.2)			
	Other	8 (7.7)	10 (9.6)			

* Italics indicate significant difference.

## Data Availability

The data that support the findings of this study are available from the corresponding author due to Ethical reason.

## References

[1] Seow W.K. (1997). Clinical diagnosis of enamel defects: Pitfalls and practical guidelines. Int. Dent. J..

[2] Nanci A. (2017). Ten Cate’s Oral Histology-E-Book: Development, Structure, and Function.

[3] Suckling G., Nelson D., Patel M. (1989). Macroscopic and scanning electron microscopic appearance and hardness values of developmental defects in human permanent tooth enamel. Adv. Dent. Res..

[4] Leal S.C., Takeshita E.M. (2019). Pediatric Restorative Dentistry.

[5] Weerheijm K.L., Mejàre I. (2003). Molar incisor hypomineralization: A questionnaire inventory of its occurrence in member countries of the European Academy of Paediatric Dentistry (EAPD). Int. J. Paediatr. Dent..

[6] Weerheijm K.L., Duggal M., Mejàre I., Papagiannoulis L., Koch G., Martens L.C., Hallonsten A.-L. (2003). Judgement criteria for Molar Incisor Hypomincralisation (MIH) in epidemiologic studies: A summary of the European meeting on MIH held in Athens, 2003. Eur. J. Paediatr. Dent..

[7] de Aguiar Grossi J., Cabral R.N., Leal S.C. (2017). Caries experience in children with and without molar-incisor Hypomineralisation: A case-control study. Caries Res..

[8] Cho S.-Y., Ki Y., Chu V. (2008). Molar incisor hypomineralization in Hong Kong Chinese children. Int. J. Paediatr. Dent..

[9] Soviero V., Haubek D., Trindade C., Da Matta T., Poulsen S. (2009). Prevalence and distribution of demarcated opacities and their sequelae in permanent 1st molars and incisors in 7 to 13-year-old Brazilian children. Acta Odontol. Scand..

[10] Zhao D., Dong B., Yu D., Ren Q., Sun Y. (2018). The prevalence of molar incisor hypomineralization: Evidence from 70 studies. Int. J. Paediatr. Dent..

[11] Mahoney E.K., Morrison D.G. (2009). The prevalence of molar-incisor hypomineralisation (MIH) in Wainuiomata children. N. Z. Dent. J..

[12] Onat H., Tosun G. (2013). Molar incisor hypomineralization. J. Pediatr. Dent..

[13] Silva M.J., Scurrah K.J., Craig J.M., Manton D.J., Kilpatrick N. (2016). Etiology of molar incisor hypomineralization—A systematic review. Community Dent. Oral Epidemiol..

[14] Bodrumlu E.H., Avşar A. (2015). Büyük azı kesici diş hipomineralizasyonu: Etiyolojisi ve kliniği. Acta Odontol. Scand..

[15] Sönmez H., Yıldırım G., Bezgin T. (2013). Putative factors associated with molar incisor hypomineralisation: An epidemiological study. Eur. Arch. Paediatr. Dent..

[16] Da Costa-Silva C.M., Jeremias F., de Souza J.F., De Cássia Loiola Cordeiro R., Santos-Pinto L., Cilense Zuanon A.C. (2010). Molar incisor hypomineralization: Prevalence, severity and clinical consequences in Brazilian children. Int. J. Paediatr. Dent..

[17] Tapias-Ledesma M.A., Jiménez R., Lamas F., González A., Carrasco P., De Miguel A.G. (2003). Factors associated with first molar dental enamel defects: A multivariate epidemiological approach. J. Dent. Child..

[18] Laisi S., Ess A., Sahlberg C., Arvio P., Lukinmaa P.-L., Alaluusua S. (2009). Amoxicillin may cause molar incisor hypomineralization. J. Dent. Res..

[19] Kühnisch J., Thiering E., Heitmüller D., Tiesler C.M., Grallert H., Heinrich-Weltzien R., Hickel R., Heinrich J., GINI-10 Plus Study Group, LISA-10 Plus Study Group (2014). Genome-wide association study (GWAS) for molar–incisor hypomineralization (MIH). Clin. Oral Investig..

[20] Ghanim A., Morgan M., Marino R., Bailey D., Manton D. (2011). Molar incisor hypomineralisation: Prevalence and defect characteristics in Iraqi children. Int. J. Paediatr. Dent..

[21] Giuca M.R., Cappe M., Carli E., Lardani L., Pasini M. (2018). Investigation of clinical characteristics and etiological factors in children with molar incisor hypomineralization. Int. J. Dent..

[22] Avery J.K., Steele P.F., Avery N. (2002). Oral Development and Histology.

[23] Caruso S., Bernardi S., Pasini M., Giuca M.R., Docimo R., Continenza M., Gatto R. (2016). The process of mineralisation in the development of human tooth. Eur. J. Paediatr. Dent..

[24] Pitiphat W., Luangchaichaweng S., Pungchanchaikul P., Angwaravong O., Chansamak N. (2014). Factors associated with molar incisor hypomineralization in T hai children. Eur. J. Oral Sci..

[25] Jälevik B., Klingberg G., Barregård L., Norén J.G. (2001). The prevalence of demarcated opacities in permanent first molars in a group of Swedish children. Acta Odontol. Scand..

[26] Whatling R., Fearne J.M. (2008). Molar incisor hypomineralization: A study of aetiological factors in a group of UK children. Int. J. Paediatr. Dent..

[27] Ghanim A., Manton D., Bailey D., Marino R., Morgan M. (2013). Risk factors in the occurrence of molar–incisor hypomineralization amongst a group of Iraqi children. Int. J. Paediatr. Dent..

[28] Allazzam S.M., Alaki S.M., El Meligy O.A.S. (2014). Molar incisor hypomineralization, prevalence, and etiology. Int. J. Dent..

[29] Bukhari S.T., Alhasan H.A., Qari M.T., Sabbagh H.J., Farsi N.M. (2023). Prevalence and risk factors of molar incisor hypomineralization in the Middle East: A systematic review and meta-analysis. J. Taibah Univ. Med. Sci..

[30] Arrow P. (2009). Risk factors in the occurrence of enamel defects of the first permanent molars among schoolchildren in Western Australia. Community Dent. Oral Epidemiol..

[31] Brogårdh Roth S., Matsson L., Klingberg G. (2011). Molar-incisor hypomineralization and oral hygiene in 10-to-12-yr-old Swedish children born preterm. Eur. J. Oral Sci..

[32] Koruyucu M., Özel S., Tuna E.B. (2018). Prevalence and etiology of molar-incisor hypomineralization (MIH) in the city of Istanbul. J. Dent. Sci..

[33] Fatturi A.L., Wambier L.M., Chibinski A.C., Assunção L.R.d.S., Brancher J.A., Reis A., Souza J.F. (2019). A systematic review and meta analysis of systemic exposure associated with molar incisor hypomineralization. Community Dent. Oral Epidemiol..

[34] Alaluusua S. (2010). Aetiology of molar-incisor hypomineralisation: A systematic review. Eur. Arch. Paediatr. Dent..

[35] Lygidakis N., Garot E., Somani C., Taylor G., Rouas P., Wong F. (2022). Best clinical practice guidance for clinicians dealing with children presenting with molar-incisor-hypomineralisation (MIH): An updated European Academy of Paediatric Dentistry policy document. Eur. Arch. Paediatr. Dent..

[36] Garot E., Rouas P., Somani C., Taylor G., Wong F., Lygidakis N. (2022). An update of the aetiological factors involved in molar incisor hypomineralisation (MIH): A systematic review and meta-analysis. Eur. Arch. Paediatr. Dent..

[37] Cutrera R., Baraldi E., Indinnimeo L., Del Giudice M.M., Piacentini G., Scaglione F., Ullmann N., Moschino L., Galdo F., Duse M. (2017). Management of acute respiratory diseases in the pediatric population: The role of oral corticosteroids. Ital. J. Pediatr..

[38] Wogelius P., Viuff J.H., Haubek D. (2020). Use of asthma drugs and prevalence of molar incisor hypomineralization. Int. J. Paediatr. Dent..

[39] Hoffmann U., Neumann C., Bauer C.-P., Berdel D., von Berg A., Koletzko S., Garcia-Godoy F., Hickel R., Heinrich J. (2014). Respiratory diseases are associated with molar-incisor hypomineralizations. Swiss Dent. J. SSO–Sci. Clin. Top..

[40] Beentjes V., Weerheijm K., Groen H. (2002). Factors involved in the aetiology of molar-incisor hypomineralisation (MIH). Eur. J. Paediatr. Dent..

[41] Kuscu O.O., Caglar E., Sandalli N. (2008). The prevalence and aetiology of molar-incisor hypomineralisation in a group of children in Istanbul. Eur. J. Paediatr. Dent..

